# Unexpected Cartilage Phenotype in CD4-Cre-Conditional SOS-Deficient Mice

**DOI:** 10.3389/fimmu.2017.00343

**Published:** 2017-03-23

**Authors:** Geoffrey Guittard, Devorah L. Gallardo, Wenmei Li, Nicolas Melis, Julian C. Lui, Robert L. Kortum, Nicholas G. Shakarishvili, Sunmee Huh, Jeffrey Baron, Roberto Weigert, Joshua A. Kramer, Lawrence E. Samelson, Connie L. Sommers

**Affiliations:** ^1^Laboratory of Cellular and Molecular Biology, CCR, NCI, NIH, Bethesda, MD, USA; ^2^Laboratory Animal Sciences Program, Leidos Biomedical Research, NCI, NIH, Bethesda, MD, USA; ^3^Section on Growth and Development, NICHD, NIH, Bethesda, MD, USA; ^4^Department of Pharmacology, Uniformed Services University of the Health Sciences, Bethesda, MD, USA

**Keywords:** SOS, RAS, proliferation, T cell signaling, cartilage homeostasis, chondrocyte dysplasia

## Abstract

RAS signaling is central to many cellular processes and SOS proteins promote RAS activation. To investigate the role of SOS proteins in T cell biology, we crossed *Sos1*^f/f^ *Sos2^−/−^* mice to CD4-Cre transgenic mice. We previously reported an effect of these mutations on T cell signaling and T cell migration. Unexpectedly, we observed nodules on the joints of greater than 90% of these mutant mice at 5 months of age, especially on the carpal joints. As the mice aged further, some also displayed joint stiffness, hind limb paralysis, and lameness. Histological analysis indicated that the abnormal growth in joints originated from dysplastic chondrocytes. Second harmonic generation imaging of the carpal nodules revealed that nodules were encased by rich collagen fibrous networks. Nodules formed in mice also deficient in RAG2, indicating that conventional T cells, which undergo rearrangement of the T cell antigen receptor, are not required for this phenotype. CD4-Cre expression in a subset of cells, either immune lineage cells (e.g., non-conventional T cells) or non-immune lineage cells (e.g., chondrocytes) likely mediates the dramatic phenotype observed in this study. Disruptions of genes in the RAS signaling pathway are especially likely to cause this phenotype. These results also serve as a cautionary tale to those intending to use CD4-Cre transgenic mice to specifically delete genes in conventional T cells.

## Introduction

The RAS/MAP kinase signaling pathway is central for cellular activation through numerous cell surface receptors. Furthermore, *RAS* is mutated in approximately 30% of all human tumors, making its study relevant to normal and cancer cell biology (1, 2). RAS can cycle between GTP- and GDP-bound forms and RAS guanine exchange factors (GEFs) facilitate conversion to the GTP-bound/active form of RAS. SOS1 is a prototypical RAS GEF. In T cells, activation through the T cell antigen receptor (TCR) results in RAS activation *via* SOS1 and other GEFs. RAS mediates downstream activation of the MAP kinases ERK1 and ERK2 (3, 4). Four RAS GEFs are active in developing and mature T cells: SOS1, SOS2, RASGRP1, and RASGRP4. Their relative expression levels determine their contributions to thymocyte development and T cell activation (3).

Genetic analysis has been essential for investigating the roles of SOS proteins in T cells. Investigation of SOS1 function was impeded when SOS1 deficiency in mice was found to be embryonic lethal (5). Kortum et al. generated floxed (conditional) *Sos1*-deficient mice to address the function of SOS1 in specific cell lineages (6). By crossing *Sos1*^f/f^ mice to Lck-Cre transgenic mice, SOS1 deficiency was achieved in developing and mature T cells. T cell development was partially disrupted. The effect on T cell development was mediated by both scaffolding and GEF functions of SOS1 (7). To bypass the effects of SOS1 on T cell development and to study the effects of SOS1 deficiency on mature T cells, we crossed *Sos1*^f/f^ mice with CD4-Cre transgenic mice (8). In addition, those mice were bred to mice deficient for the related *Sos* family member SOS2 (9). *Sos1*^f/f^ *Sos2^−/−^* CD4-Cre^+^ mice had normal numbers and subsets of T cells. T cells from *Sos1*^f/f^ *Sos2^−/−^* CD4-Cre^+^ mice had intact ERK activation downstream from the TCR but defective ERK activation downstream from the IL-2 receptor. The *Sos1*^f/f^ *Sos2^−/−^* CD4-Cre^+^ mice displayed defects in T cell migration that were secondary to increased PI3kinase activity in T cells (8).

For the latter studies, CD4-Cre was chosen for conditional knockout of *Sos1* because it is expressed later in thymocyte development than Lck-Cre (10). Lck-Cre is expressed at the early CD4 CD8 double negative stage whereas CD4-Cre is expressed at the transitional CD4 CD8 double positive stage. CD4-Cre is widely used for conditional deletion of genes in double positive and mature T cells as the original description of CD4-Cre (11) has been cited over 500 times. Recently the integration of the CD4-Cre transgene, which expresses the Cre recombinase gene under the control of the promoter and regulatory elements of the *Cd4* gene, has been mapped to mouse chromosome 3 and is present in at least 15 copies (12).

In addition to the signaling effects described above that we observed in mature T cells from *Sos1*^f/f^ *Sos2^−/−^* CD4-Cre^+^ mice, we also observed that the mice developed nodules on multiple joints as they aged, especially carpal joints, and could eventually develop hind limb paralysis and become lame. We postulate that these disabling defects arise from abnormal cartilage homeostasis. Our unexpected results of a cartilage-based phenotype in *Sos1*^f/f^ *Sos2^−/−^* CD4-Cre^+^ mice provide evidence that CD4-Cre expression may not be restricted to conventional double positive and mature CD4^+^ and CD8^+^ T cells.

## Materials and Methods

### Mice

*Sos1*^f/f^ and *Sos2^−/−^* mice were previously described (6, 9). CD4-Cre transgenic mice were from Taconic (Hudson, NY, USA). *Rag2*-deficient mice were from Jackson Laboratory (Bar Harbor, ME, USA). All mice were on a C57BL/6 background. Mice were housed at the National Cancer Institute (NCI). This study was carried out in accordance with guidelines set forth by the NCI-Bethesda Animal Care and Use Committee. The protocol was approved by the NCI-Bethesda Animal Care and Use Committee.

### Clinical Scoring

**Figure 1:**
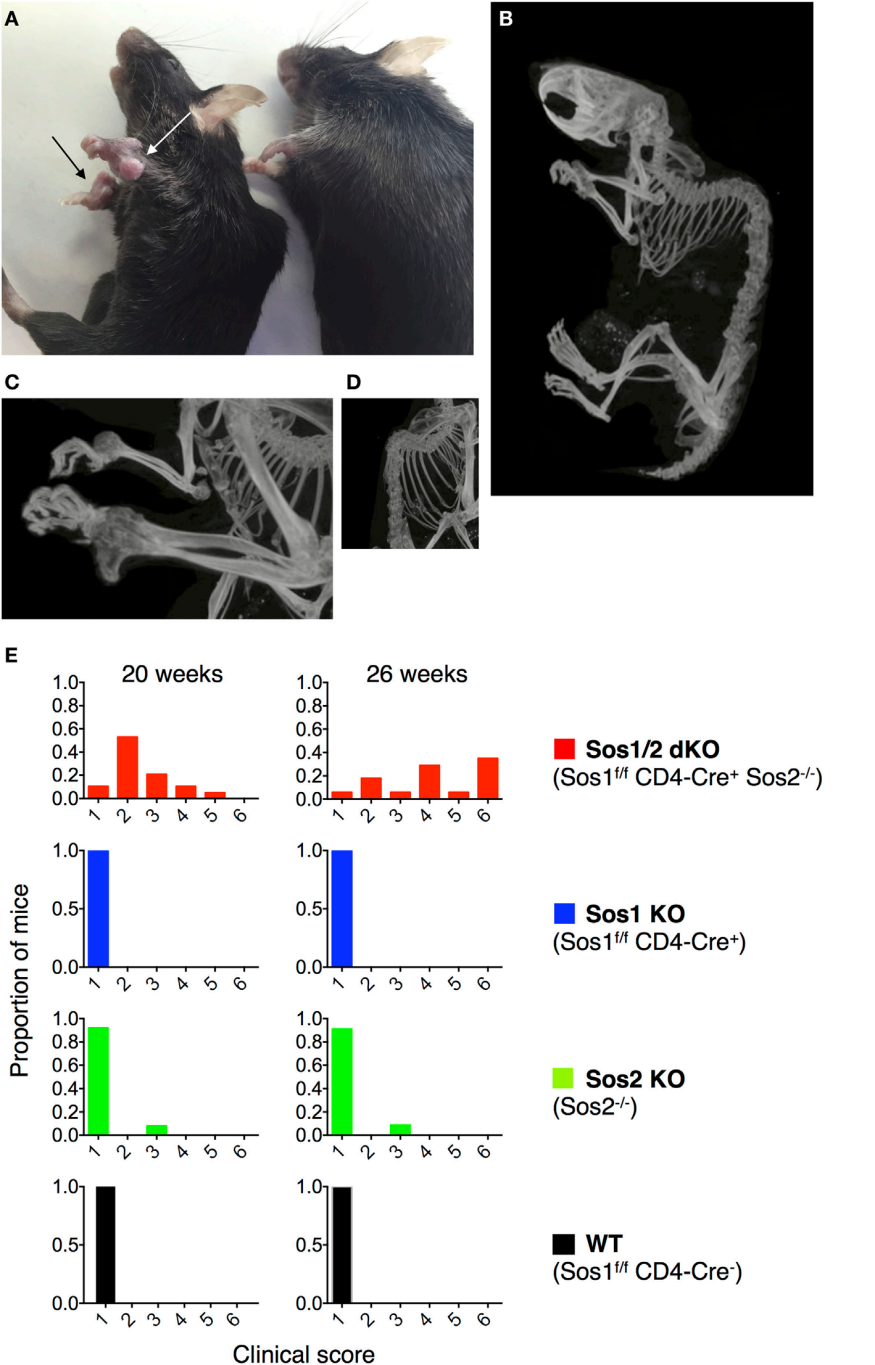
**Joint abnormalities in Sos1/2 dKO mice**. **(A)** Nodules are evident on the carpal joints of a 45-week-old Sos1/2 dKO mouse (left, arrows) and not on a 45-week-old Sos2 KO mouse (right). **(B–D)** Computerized tomographic (CT) images of 32-week-old Sos1/2 dKO mouse. Videos S1 and S2 in Supplementary Material contain 3D video images of Sos1/2 dKO and control Sos2 KO CT scanning. **(E)** The four genotypes under study are listed with their color codes. A clinical score was determined for individual mice at 20 and 26 weeks of age. The clinical scores were based on the presence of nodules on joints, joint flexibility, and gait. A score of 1 indicates no nodules on joints, normal joint flexibility, and normal gait. A score of 2 indicates nodules visible on joints upon close inspection, normal joint flexibility, and normal gait. A score of 3 indicates clearly visible nodules on joints, normal joint flexibility, and slightly abnormal gait. A score of 4 indicates nodules visible cageside on multiple joints, reduced joint flexibility, and abnormal gait visible cageside. A score of 5 indicates nodules visible cageside on multiple joints, severely reduced joint flexibility, and reduced movement in a foreign area (severely lame). A score of 6 indicates nodules visible cageside on multiple joints, severely reduced joint flexibility with no flexibility in hind limbs, and no movement in a foreign area (extremely lame). For WT, *n* = 8 at 20 weeks and 7 at 26 weeks; for Sos1 KO, *n* = 13; for Sos2 KO, *n* = 13 at 20 weeks and 11 at 26 weeks; for Sos1/2 dKO, *n* = 19 at 20 weeks and 18 at 26 weeks.

Mice were scored using the parameters listed in Figure 1E legend by an observer who was blind to the genotypes of the mice. Mice with high clinical scores were fed with gelmeal inside the cage in cases where it was suspected that the mice might have difficulty reaching food.

### Computerized Tomographic (CT) Imaging

Mice were euthanized and fixed in neutral buffered formalin for 24 h. Then, mice were preserved in 70% ethanol prior to imaging. 3D images were acquired on a CT animal scanner with a resolution of 9 μm. CT imaging was performed by the Mouse Imaging Facility of the NIH *In Vivo* NMR Center.

### Histology

Sections of bone were decalcified, embedded in paraffin, sectioned at 5 μm, and stained with H&E and Toluidine blue using standard techniques by the Pathology/Histotechnology Laboratory of the Laboratory Animal Sciences Program at NCI-Frederick. Stained slides were scanned into a digital format an Aperio Scanscope XT (Leica, Vista, CA, USA) at 20× magnification. Images were captured using Aperio ImageScope v12.2.2.5015.

### Second Harmonic Generation (SHG) Imaging

The carpal nodules and control areas were excised from the mice, placed on a coverglass, and imaged by SHG, using an inverted laser scanning two-photon microscope (MPE-RS, Olympus, Center Valley, PA, USA) equipped with a tunable laser (Insight DS+, Spectra Physics, Santa Clara, CA, USA). Samples were excited at 900 nm and the SHG signal (450 nm) was collected on a GaAs detector using a dichroic mirror (SDM570) and a bandpass filter (BP/410-470). Low magnification images were acquired using a 4× air objective [UPLSAPO4X(F), Olympus] whereas high magnification images were acquired with a silicon oil immersion 30× objective (UPLSAPO30XIR, Olympus).

### Purification of CD4^+^ T Cells/DNA Isolation/Genomic PCR

CD4^+^ T cells were isolated from lymph nodes and spleens of *Sos1*^f/f^ *Sos2^−/−^* CD4-Cre^+^ mice using EasySep Mouse CD4+ T Cell Isolation Kit (Stem Cell Technologies, Cambridge, MA, USA). Purity was >80%. Non-lymphoid tissues were homogenized prior to DNA isolation using a TissueMiser System, 115 V (Fisher Scientific, Hampton, NH, USA). Tissue DNA was isolated using DNA columns from a ZR-Duet DNA/RNA MiniPrep Plus kit (Zymo Research, Irvine, CA, USA). Genotyping PCRs used for tissue DNAs were described previously (6).

### RNA Isolation from Growth Plate

Proximal tibias were dissected from 1-week-old wild-type C57BL/6 male mice and, from them, the growth plate cartilage in the center was isolated by removing the metaphyseal bone, perichondrium, meniscus, and articular cartilage. The growth plate cartilage was homogenized and RNA isolated using an RNeasy mini kit following the manufacturer’s instructions (Qiagen). RNA integrity was determined using an Agilent 2100 Bioanalyzer (Agilent Technologies, Santa Clara, CA, USA) and only high quality RNA (RIN > 7) was used for real-time RT-PCR.

### Quantitative Real-time RT-PCR

Real-time PCR was used to compare gene expression in CD4^+^ T cells purified as described above from C57BL/6 lymph nodes in single cell suspensions from C57BL/6 lymph nodes and in growth plate chondrocytes. Total RNA (500 ng) was reverse-transcribed using SuperScript IV Reverse Transcriptase (Thermo Scientific). Quantitative real-time PCR was performed for *18S, Sos1, Cd4, Cd3e*, and *Col2a1* using commercially available FAM or VIC-labeled Taqman assays (Thermo Scientific). All assays or primer pairs were intron-spanning to avoid amplification of genomic DNA. Reactions were performed in triplicate using the ViiA 7 Real-time PCR System (Thermo Scientific). The relative quantity of each mRNA was calculated using the formula: relative expression = 2^−ΔCt^ × 10^8^, where Ct represents the threshold cycle and ΔCt = (Ct of gene of interest) − (Ct of 18S rRNA). Values were multiplied by 10^8^ for convenience of comparison.

## Results

To investigate the effects of *Sos1* and *Sos2* deletion in T cells, we crossed Sos1^f/f^ *Sos2^−/−^* mice to CD4-Cre transgenic mice. Expression of Cre recombinase in CD4 CD8 double positive thymocytes, in single positive thymocytes, and in mature CD4^+^ and CD8^+^ T cells results in an absence of SOS protein expression in these cells, which is consequent to the deletion of *Sos1* exon 10 by Cre recombinase (6). We were surprised, then, to observe that *Sos1*^f/f^ *Sos2^−/−^* CD4-Cre^+^ mice developed visible nodules on multiple joints, especially carpal joints (Figure 1A, arrows) and had reduced joint flexibility by about 16 weeks of age. 3D CT imaging of a 32-week-old *Sos1*^f/f^ *Sos2^−/−^* CD4-Cre^+^ mouse showed bony protrusions on the carpal joints and severe kyphosis of the spine (Figures 1B–D; Videos S1 and S2 in Supplementary Material). As the mice aged, some exhibited hind limb paralysis and became lame. Figure 1E shows clinical scoring based on nodule presence on joints, joint flexibility, and gait. An observer blinded to the genotypes scored the mice at 20 and 26 weeks of age. The observer examined the following four genotypes: WT (*Sos1*^f/f^ *Sos2*^+/+^ CD4Cre^−^), Sos1 KO (*Sos1*^f/f^ *Sos2*^+/+^ CD4Cre^+^), Sos2 KO (*Sos1*^f/f^ *Sos2^−/−^* CD4Cre^−^), and Sos1/2 dKO (*Sos1*^f/f^ *Sos2^−/−^* CD4Cre^+^). With the exception of one Sos2 KO mouse, the only mice that scored above a normal clinical score of 1 were Sos1/2 dKO mice. At 26 weeks of age, 16 of 17 Sos1/2 dKO mice had measurable clinical manifestations of disease and 12 of 17 Sos1/2 dKO mice had severely reduced joint flexibility and were lame (clinical score greater than or equal to 4).

**Figure 2:**
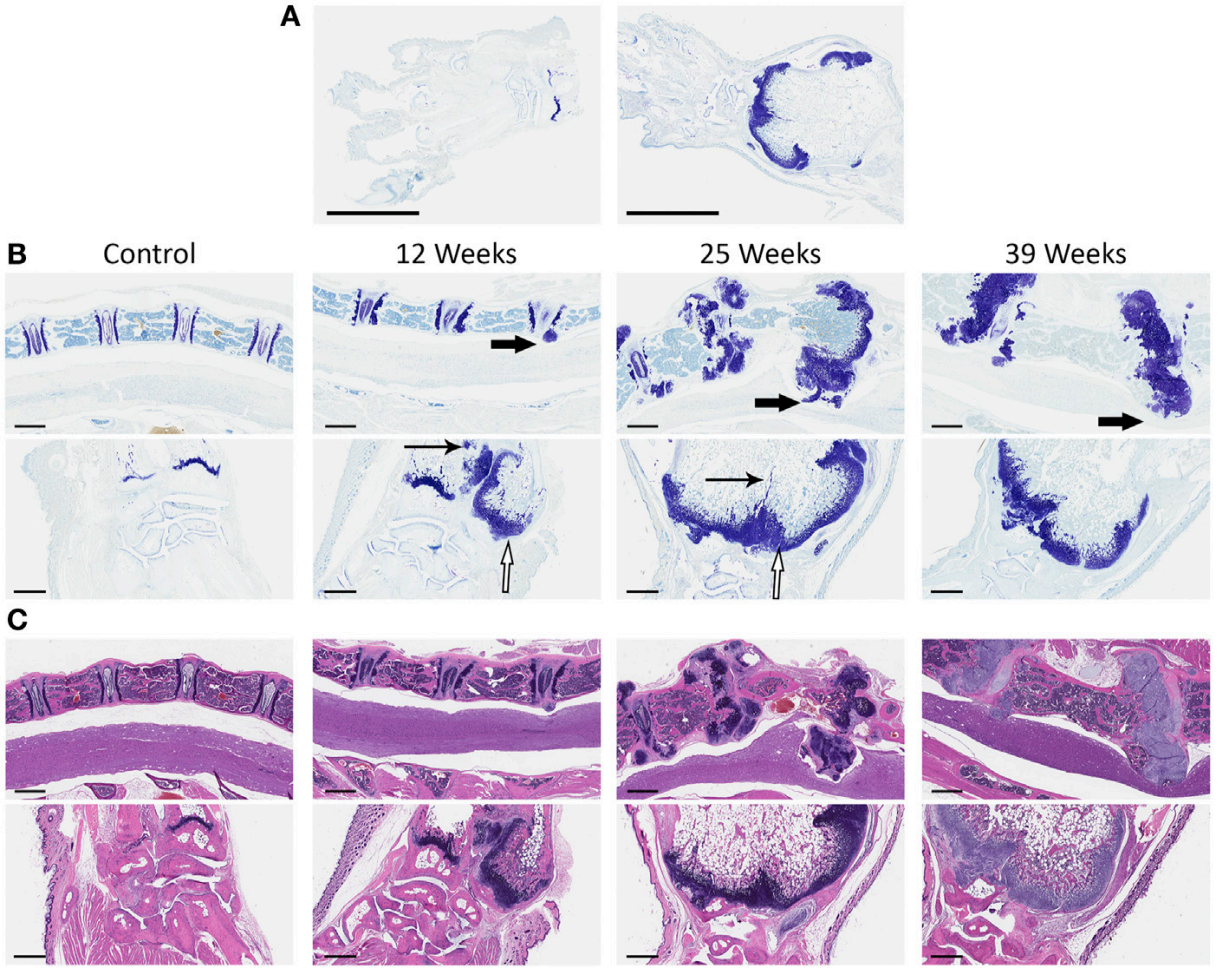
**Chondrocyte dysplasia in Sos1/2 dKO joints**. **(A)** At low magnification, control Sos2KO (left) versus Sos1/2 dKO (right) showed clear differences following toluidine blue staining. The radial physis of Sos1/2 dKO animals is expanded by chondrocytes and the medullary cavity is enlarged significantly (scalebar = 3 mm). **(B)** Vertebrae (top panels, 4× magnification, scalebar = 600 μm) and carpal joints (bottom panels, 5× magnification, scalebar = 500 μm) were sampled at various times and stained with toluidine blue to compare the changes in affected joints over time. **(C)** H&E staining of vertebrae (top) and carpal joints (bottom) as in **(B)**. Control Sos2 KO mice had normal vertebrae and carpal joints at all time points. Beginning at 12 weeks of age, proliferation of dysplastic chondrocytes was seen at growth plates of Sos1/2 dKO mice, and these changes became more pronounced over time. There was prominent, irregular thickening of the physes (hollow arrows) and multifocal areas of unresorbed cartilage in the metaphyses (thin arrows). The proliferations of dysplastic chondrocytes, both within the growth plate and protruding from it, impinged on adjacent structures including the spinal cord (thick arrows), likely contributing to the secondary clinical signs seen in these animals including hind limb paresis and altered gait.

To further characterize the abnormal structures that we observed clinically, we performed histological analysis on the vertebrae, carpal joints, and stifle joints of Sos1/2 dKO and control Sos2 KO mice. Figure 2 shows Hematoxylin and Eosin staining and Toluidine blue staining of vertebrae and carpal joints. Toluidine blue staining, which preferentially stains cartilage blue, showed increased amounts of abnormally organized cartilage in these joints in Sos1/2 dKO mice, but not Sos2 KO mice. By 12 weeks of age, the growth plates of Sos1/2 dKO mice showed proliferation of dysplastic chondrocytes and these changes became more prominent over time. By 25 and 39 weeks, dysplastic chondrocyte proliferation had resulted in irregular and sometimes severe thickening of the physes that multifocally impinged on adjacent structures, including the spinal cord. Spinal cord compression likely contributed to the hind limb paresis and altered gait seen clinically in these mice. Importantly, there was no evidence of lymphocyte infiltration or inflammation in Sos1/2 dKO sections.

**Figure 3:**
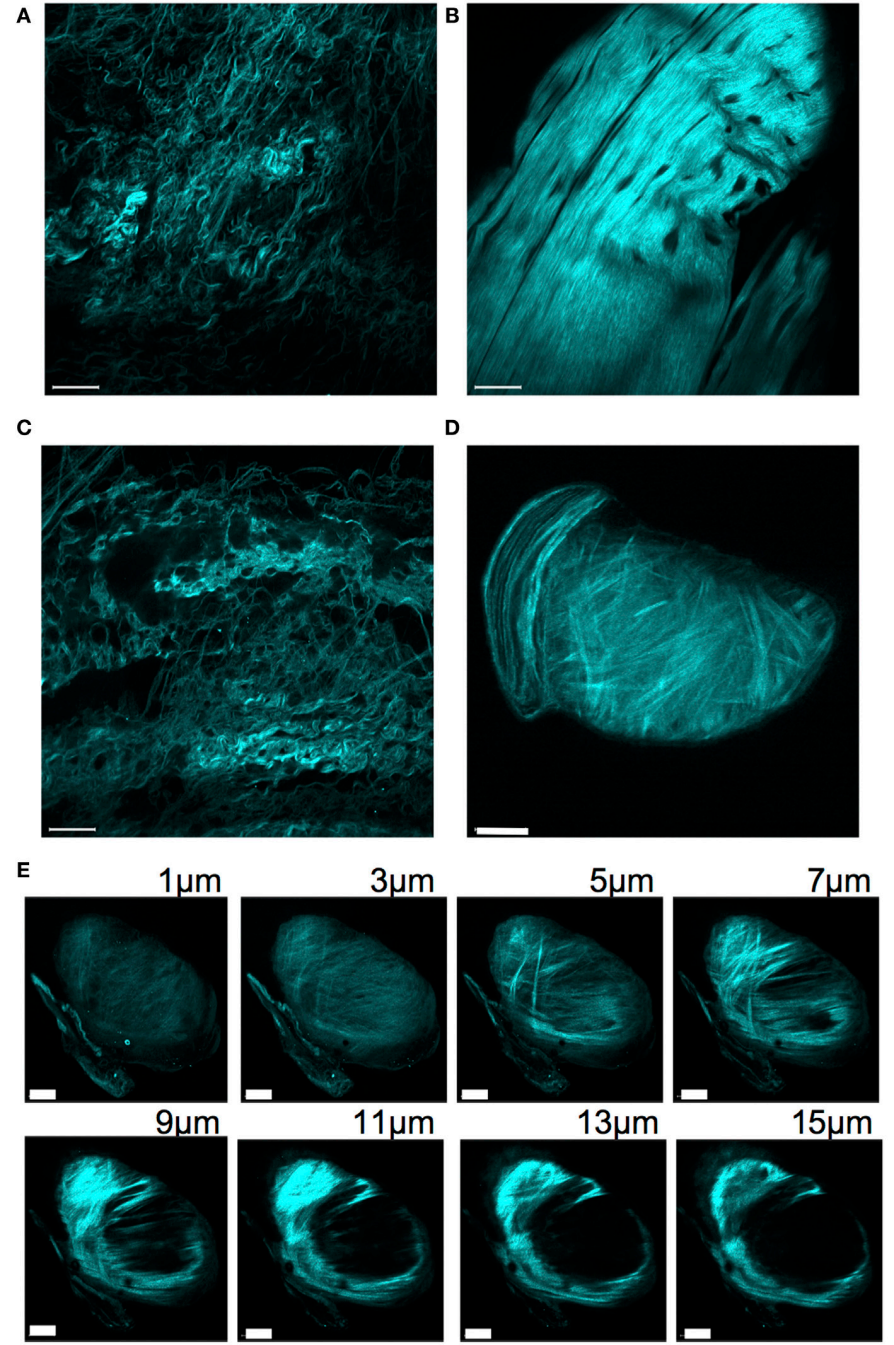
**Second harmonic generation of collagen network surrounding carpal joints**. Tissue surrounding carpal joints was excited at 900 nm and backscattered emissions collected at 410–470 nm to reveal the SHG signal. **(A)** Sos2 KO (32 weeks), **(B)** Sos1/2 dKO (32 weeks), **(C)** WT (48 weeks), **(D)** Sos1/2 dKO (48 weeks). Scale bar = 50 μm. **(E)** Intensity *Z*-stack of a carpal joint nodule for the mouse used in panel **(D)**. Scale bar = 40 μm.

To better understand the tissue architecture of the carpal nodules, we performed SHG imaging of Sos1/2 dKO carpal nodules and the corresponding area of control Sos2 KO carpal joints. SHG enables the label-free visualization of collagen fibers in various tissues, as previously shown (13). A loosely organized pattern of wavy collage fibrils was observed in control carpal joints (Figures 3A,C). In contrast, the pattern in the Sos1/2 dKO carpal nodules contained relatively straight collagen fibrils that were closely aligned (Figures 3B,D). SHG imaging of edges of the nodules indicated that the nodules seemed to be encased in a rich collagen network (Figure 3E).

**Figure 4:**
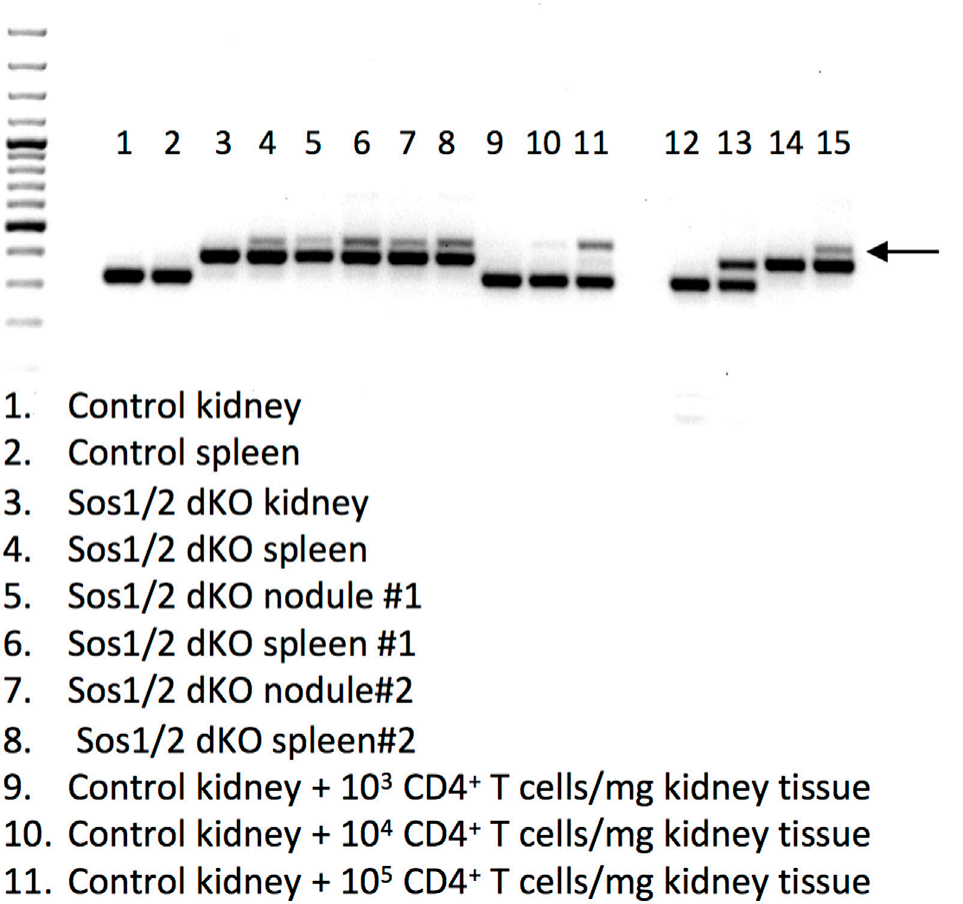
***Sos1* exon 10 excision in tissues from Sos1/2 dKO mice**. A 3 primer PCR was performed to yield wild-type *Sos1* bands (bottom bands), floxed *Sos1* (middle bands), and deleted *Sos1* (upper bands, arrow). Lanes: 1–11 as indicated in figure, (12) *Sos1*^+/+^ control, (13) *Sos1*^+/f^ control, (14) *Sos1*^f/f^ control, (15) *Sos1*^f/f^ + Cre control. For lanes 1 and 2, a 35-week-old C57BL/6 mouse was used. For lanes 3 and 4, a 34-week-old, for lanes 5 and 6, a 36-week-old, and for lanes 7 and 8, a 22-week-old Sos1/2 dKO was used.

Because T cell infiltration was not seen histologically in carpal nodule sections (Figure 2C), we postulated that some other non-T cells in the Sos1/2 dKO nodules might have deleted *Sos1* exon 10. To investigate *Sos1* deletion in carpal nodules, we homogenized nodule tissue and performed a genomic PCR that distinguishes between wild-type *Sos1*, floxed *Sos1*, and deleted *Sos1* (Figure 4). Lanes 5 and 7 show that nodule tissue does indeed contain cells with *Sos1* exon 10 deletion. We also spiked normal tissue with known numbers of mature, deleted CD4^+^ T cells derived from the lymphoid organs of Sos1/2 dKO mice. These samples showed that greater than 10^4^ T cells/mg tissue would be required to see a deleted signal such as that seen in lanes 5 and 7. Examination of the pathology of Sos1/2 dKO carpal tissue indicates that it is extremely unlikely that the deleted PCR band originating from carpal tissue arises exclusively from T cells because lymphocyte infiltration was not observed in nodule sections.

To investigate whether normal chondrocytes express *Cd4* and, therefore, might be susceptible to CD4-Cre-mediated deletion, we performed quantitative real-time RT-PCR on growth plate cartilage from 1-week-old wild-type mice (Table 1). Levels of *Cd4* expression were extremely low compared to the positive controls from lymph node single cell suspensions and purified CD4^+^ lymph node T cells. However, the levels of *Cd4* expression in growth plate cartilage were slightly higher than *Cd3e* levels.

**Table 1:** **Relative expression^a^ of Cd4 in wild-type growth plate cartilage**.

Tissue	Cd3e	Cd4	Sos1	Col2a1
Lymph node	67,775	8,388	1,961	0
CD4^+^ T cells	166,058	37,825	3,084	0
Growth plate 1	4	19	431	388,222
Growth plate 2	5	40	623	430,022

*^a^Relative expression levels were determined by quantitative real-time RT-PCR as described in Section “Materials and Methods.” Growth plates 1 and 2 are biological replicates*.

To more definitively determine if mature, conventional CD4^+^ and CD8^+^ T cells, which are the usual intended target of CD4-Cre transgenesis, were responsible for cartilage disease and nodule formation in Sos1/2 dKO mice, we crossed Sos1/2 dKO mice to mice deficient in RAG2, a recombinase that is required for the formation of mature T and B lymphocytes. As shown in Table 2, all Sos1/2 dKO *Rag2^−/−^* mice developed carpal nodules at 18–20 weeks similar to Sos1/2 dKO *Rag2*^+/+^ mice. At 26 weeks, 6 of 6 Sos1/2 dKO *Rag2^−/−^* mice had a maximum clinical score of 6 whereas 2 of 4 Sos1/2 dKO *Rag2*^+/+^ mice had a clinical score of 6. These data indicate that mature, conventional TCR-expressing T cells are not required for development of the cartilage-based disease described in this study. In fact, in the absence of conventional T and B lymphocytes, clinical disease was more severe.

**Table 2:** **Sos1/2 dKO clinical disease is not dependent on conventional T cells**.

Genotype	Number of mice with clinical score >1 at 18–20 weeks of age^a^	Number of mice with clinical score = 6 at 26 weeks of age^a^
Rag2^+/+^ Sos1/2 dKO	3/4	2/4
Rag2^−/−^ Sos1/2 dKO	5/5	6/6

*^a^Clinical scores were determined by an observer blinded to genotype. The clinical scoring method is described in the legend of Figure 1*.

## Discussion

To investigate the role of SOS proteins in T cells, we crossed *Sos1*^f/f^ *Sos2^−/−^* mice with CD4-Cre transgenic mice. We previously reported no effect on T cell numbers, but an effect of these mutations on T cell signaling and T cell migration (8). Unexpectedly, as the dKO mice aged, we observed nodules in the joints of greater than 90% of these mutant mice, especially at carpal joints. Histological analysis suggested that the abnormal growth originated from dysplastic chondrocytes. An important question is whether the joint abnormality that we observed is caused by T cells, as would be expected from using CD4-Cre, or by other non-T cells expressing CD4-Cre sometime in their lifetime. Histological analysis did not show lymphocytic infiltrates in the nodules. Furthermore, crosses to *Rag2^−/−^* mice indicated that the phenotype was not caused by T cells, at least not conventional T cells that undergo rearrangement of the TCR, because nodules still formed in the joints of the Sos1/2 dKO *Rag2^−/−^* mice.

What is the origin of the chondrocyte dysplasia in Sos1/2 dKO mice? One possible scenario is that the cartilage nodules arise from impaired SOS/RAS/MAPK signaling in a rare subset of chondrocytes consequent to CD4-Cre expression in those chondrocytes. Analysis of *Cd4* RNA expression in normal chondrocytes from growth plate cartilage showed low, but detectable, expression leaving open the possibility that CD4-Cre is active in a small subset of chondrocytes. This scenario is consistent with previous studies demonstrating that the RAS signaling pathway, mediated by SOS, inhibits chondrocyte proliferation (14). In chondrocytes, a fibroblast growth factor, FGFR3, signals through SOS to activate the RAS/MAPK pathway. Downstream from RAS, RAF activates MEK1/2 and MEK5/6, which activate ERK and p38, respectively. Postnatal chondrocyte-specific *Fgfr3* deletion results in chondroma-like lesions (15). Other RAS/MAPK perturbations that result in bone growth abnormalities include constitutively active MEK1 (16) and conditional inactivation of *Erk1* and *Erk2* in chondrocytes (17). Similarly, conditional knockout of *Shp2* in chondrocytes results in the formation of cartilaginous nodules, similar to the phenotype seen in the current study. SHP2 is a protein tyrosine phosphatase that mediates RAS/MAPK signaling downstream from many growth factor receptors including FGFR (18, 19).

In humans, patients with Noonan syndrome have inherited mutations in RAS/MAPK pathway genes. Noonan syndrome is a multisystem and varied disease often characterized by distinctive facial features, developmental delay, short stature, congenital heart disease, and other abnormalities (20). About 10% of Noonan syndrome patients have *SOS1* mutations that are thought to confer hyperactivity of SOS1. The short stature in these patients suggests that activating mutations in *SOS1* impair growth plate chondrogenesis, whereas in our mouse model, loss of SOS1 and SOS2 appears to increase chondrogenesis.

Although it is possible that some rare chondrocytes express *Cd4* and that chondrocyte dysplasia results from SOS deficiency in those chondrocytes, it is also possible that conventional T cells regulate chondrocyte proliferation and that SOS deficiency in T cells results in alterations in secretion of factors that normally keep chondrocyte proliferation in check. This cannot be the only mechanism at play, however, because *Rag2^−/−^* mice (with wild-type SOS) do not have conventional T cells and do not exhibit a cartilage phenotype. Another possible scenario is that immune regulatory cells, which express *Cd4* sometime in their lifetime, but are not conventional T or B lymphocytes, keep chondrocyte proliferation in check. Consequent to SOS deficiency in these cells, chondrocyte dysplasia would result, disturbing cartilage homeostasis. Our results that 26-week-old Sos1/2 dKO *Rag2^−/−^* mice have more severe disease than Sos1/2 dKO *Rag2*^+/+^ mice could indicate that imbalances in immune cell subpopulations can alter cartilage homeostasis. At the least, these results imply that the disease is not completely due to a chondrocyte effect. Some combination of these possible causes of chondrocyte dysplasia, SOS deficiency in rare chondrocyte populations, in T cells, or in regulatory non-T cells, may be responsible for development of this clinical disease. Control of cartilage homeostasis by T cells and/or by hypothetical immune, non-T cells would reveal a novel function for the immune system.

The extensive prior evidence that SOS/RAS/MAPK pathway signaling inhibits chondrogenesis provides for the possibility that *Sos1* is excised by CD4-Cre in occasional chondrocytes or chondrocyte precursors. In combination with the germline loss of *Sos2*, RAS/MAPK signaling could be impaired, stimulating chondrocyte proliferation and nodule formation. Alternatively, *Sos1* could be deleted in other cell types that regulate chondrogenesis *via* the secretion of cytokines or growth factors. In accordance with the hypothesis that CD4-Cre-regulated deletion of SOS/RAS/MAPK pathway genes can lead to the cartilage-based phenotype described here, the laboratory of Maureen McGargill has found that mice with both a germline mutation of *Erk1* and conditional deletion of *Erk2* mediated by CD4-Cre exhibit a similar disease to that described in this study (Marie Wehenkel and Maureen McGargill, St. Jude Children’s Research Hospital, Memphis, TN, USA, personal communication). As ERK kinases are MAPKs activated downstream from SOS proteins, it is extremely likely that the SOS/RAS/MAPK pathway causes the phenotype described here. Our results serve as a cautionary tale to those intending to use CD4-Cre transgenic mice. In addition to being expressed in conventional T cells, CD4-Cre may be expressed in a subset of non-T cells, either immune lineage cells (e.g., non-conventional T cells) or non-immune lineage cells (e.g., chondrocytes). This expression may give rise to the dramatic phenotype observed here, depending on the gene being deleted by the Cre recombinase. Disruptions of genes in the RAS signaling pathway are particularly likely to render this phenotype.

## Author Contributions

GG, JB, RW, JK, LS, and CS designed the study. GG, DG, WL, NM, JL, RK, NS, SH, and CS performed the research. JB, RW, JK, LS, and CS supervised the study. CS, GG, JB, RW, JK, and LS wrote the paper.

## Conflict of Interest Statement

The authors declare that the research was conducted in the absence of any commercial or financial relationships that could be construed as a potential conflict of interest.

## Supplementary Material

Video S1 **3D CT imaging of 32-week-old Sos1/2 dKO mouse**.

Video S2 **3D CT imaging of 32-week-old control Sos2 KO mouse**.
